# The Development of Appetite: Tracking and Age-Related Differences in Appetitive Traits in Childhood

**DOI:** 10.3390/nu15061377

**Published:** 2023-03-12

**Authors:** Elena Jansen, Gita Thapaliya, Jennifer Beauchemin, Viren D’Sa, Sean Deoni, Susan Carnell

**Affiliations:** 1 Division of Child & Adolescent Psychiatry, Department of Psychiatry & Behavioral Sciences, Johns Hopkins University School of Medicine, Baltimore, MD 21287, USA; 2 Advanced Baby Imaging Lab, Brown University & Hasbro Children’s Hospital, Rhode Island Hospital, 593 Eddy Street, Providence, RI 02903, USA; 3 Bill & Melinda Gates Foundation, 500 5th Ave North, Seattle, WA 98109, USA

**Keywords:** appetite, tracking, age-related differences, development, child

## Abstract

Appetitive traits are associated with body weight. Increased understanding of how appetitive traits evolve from early life could advance research on obesity risk and inform intervention development. We report on tracking and age-related differences in appetitive traits in childhood within the RESONANCE cohort. Parents of RESONANCE children aged 6.02 ± 2.99 years completed the Child Eating Behavior Questionnaire (CEBQ). Pearson correlations of appetitive traits and age were tested for all participants contributing at least one observation, using each participant’s first observation (N = 335). Children’s first and second observations of the CEBQ (n = 127) were used to test tracking (paired correlations) and age-related differences (paired *t*-tests) within individuals. CEBQ correlations with age suggested that satiety responsiveness, slowness in eating, emotional undereating, and desire to drink decreased with age (r = −0.111 to r = −0.269, all *p* < 0.05), while emotional overeating increased with age (r = 0.207, *p* < 0.001). Food fussiness demonstrated a quadratic relationship with age. Paired *t*-tests further supported an increase in emotional overeating with age (M: 1.55 vs. 1.69, *p* = 0.005). All CEBQ subscales demonstrated moderate to high tracking (r = 0.533 to r = 0.760, *p* < 0.001). Our initial findings within the RESONANCE cohort suggest that food avoidant traits are negatively related with age, while emotional overeating increases with age, and that appetitive traits track through childhood.

## 1. Introduction

Appetitive traits are dispositions toward food that differ between individuals [1] and show associations with body weight in childhood [2]. A key proposition of the behavioral susceptibility theory of child obesity [3] is that even as children age and start to consume different types of food (e.g., milk to table foods) and eat in different contexts (e.g., with peers, at school), appetitive traits persist. That is, if a child shows a more avid appetite and a greater interest in food in infancy, similar food approach tendencies are expected as the child grows up. However, although tracking of body weight through childhood is well-established [4], relatively few studies have directly examined the question of whether child appetite tracks, i.e., whether children maintain their relative levels of appetite in relation to their peers across development. Moreover, few studies, including those examining tracking, have described developmental changes in appetitive traits, i.e., the ways in which appetitive traits change with age across the population. Answering these questions is important because an understanding of normative appetite development is essential to interpret the meaning of observed individual differences. For example, if a child or group of children has different appetitive trait scores to a comparison group or the same individual/s at a different age, are these levels consistent or inconsistent with what one might expect based on extant data? Clarity on normative developmental trajectories of child appetite could also inform interventions to prevent or treat obesity by addressing eating behavior. For example, a deeper understanding of tracking and developmental trends could aid in the identification of critical windows for population intervention. In addition, personalized information for parents about their child’s appetite and expected trajectories could be used to develop tailored advice and support for responsive feeding practices. Starting these interventions early is essential, since a recent study found that eating behaviors in childhood have long-lasting influences on not only diet and weight status, but also eating behaviors in adults [5]. Appetitive traits in adults, in turn, have been found to be associated with body weight [6,7]. For example, one recent study of a UK cohort using the Adult Eating Behavior Questionnaire (AEBQ) demonstrated that food responsiveness, enjoyment of food, and emotional overeating were positively associated with BMI, while satiety responsiveness, emotional undereating, and slowness in eating were negatively associated with BMI [6]. Similar findings were obtained in a study of an Australian sample, which found that emotional overeating was positively associated with BMI, while satiety responsiveness and slowness in eating were negatively associated with BMI [7].

Two early studies from the UK including 322 children at ages 4 and 11 years [8] and 31 children at ages 2 and 5 years, respectively [9], used the Child Eating Behavior Questionnaire (CEBQ) [10] to investigate the stability (i.e., tracking within individuals; same rank order over time) and discontinuity (i.e., age-related differences within individuals) of appetitive traits during childhood. Both studies found evidence for stability across all subscales (with the exception of enjoyment of food in the latter study) but differed in their findings relating to (dis-)continuity. Ashcroft et al. [8] found that all measured scales changed from age 4 to 11 years (i.e., decrease in satiety responsiveness, slowness in eating, food fussiness, and emotional undereating and increase in food responsiveness, enjoyment of food, and emotional overeating; desire to drink was not assessed), while Farrow et al. [9] found a decrease in desire to drink from 2 to 5 years but no changes in food responsiveness, emotional overeating, enjoyment of food, satiety responsiveness, slowness in eating, emotional undereating, or food fussiness. Similar findings have more recently emerged from prospective or cross-lag studies with 800 to 3800 children from Europe, such that tracking has been observed for all scales across the different time lags (e.g., 7 to 10 years [11], 4 to 10 years [12], and 6 to 8 to 10 years [13]). We are aware of fewer studies that both report and statistically compare paired mean scores across different child ages. Costa et al. [11], similar to Ashcroft, found that most traits changed between 7 and 10 years of age (i.e., increase in food responsiveness, enjoyment of food, and emotional overeating; decrease in satiety responsiveness, slowness in eating, and emotional undereating, and also in desire to drink), with no change in food fussiness. The aim of the current study was to build on the above findings by investigating how appetitive characteristics evolve during childhood via the RESONANCE study, a US cohort including children aged 2 to 15 years. Due to the wide age range of the cohort, we were able to explore cross-sectional relationships of all Child Eating Behavior Questionnaire scales with age through early to later development, in addition to conducting analyses of change and tracking within subjects over a relatively narrow timespan.

## 2. Materials and Methods

### 2.1. Study Sample

The data used in the present study came from RESONANCE, a large ongoing cohort study of socioeconomically diverse mother–child dyads beginning in infancy with a focus on early brain development [14,15]. RESONANCE is part of the NIH-funded ECHO program (http://echochildren.org (accessed on 24 January 2023)). Children (983 currently active) are followed longitudinally, with study visits every 6 (biannually up to age 24 months) or 12 months (annually after age 24 months). Participants were recruited either during pregnancy or when children were between the ages of birth and 5 years old. A variety of methods was used, including flyers; Facebook and social media; radio advertisements; community events; and in-person information sessions at school, daycares, hospitals, and community centers. Infants and children with known risk factors for learning and/or psychiatric disorders were excluded (e.g., birth prior to 32 weeks’ gestation or birthweight < 1500 g, non-singleton or complicated pregnancy, neurological trauma in child, psychiatric history in a parent or sibling) [16]. For the current study, all participants with at least one assessment of appetitive characteristics in early childhood were included. For the longitudinal data analysis, participants with two or more observations were selected. For the present paper, only the first and second observations (i.e., assessments of appetitive traits) for the Child Eating Behavior Questionnaire were evaluated. Written consent was obtained from parents or legal guardians in accordance with ethics approval from the host institution’s Institutional Review Board (IRB no.: 1500991).

### 2.2. Measurement Tools

#### 2.2.1. Child Eating Behavior

To assess appetitive traits in children consuming solid foods, parents of children aged 2 years and older completed the Child Eating Behavior Questionnaire (CEBQ) [10]. The Child Eating Behavior Questionnaire consists of 35 items measured on a Likert-type scale (1 = “never” to 5 = “always”) and comprises eight subscales assessing food approach behaviors, including food responsiveness (FR, example item 1: “Even if my child is full s/he finds room to eat his/her favorite food”, example item 2: “If given the chance, my child would always have food in his/her mouth”), enjoyment of food (EF, example item 1: “My child looks forward to mealtimes”, example item 2: “My child is interested in food”), emotional overeating (EOE, example item 1: “My child eats more when worried”, example item 2: “My child eats more when s/he has nothing else to do”), and desire to drink (DD, example item 1: “If given the chance, my child would drink continuously throughout the day”, example item 2: “If given the chance, my child would always be having a drink”), and food avoidant behaviors, including satiety responsiveness (SR, example item 1: “My child leaves food on his/her plate at the end of a meal”, example item 2: “My child cannot eat a meal if s/he has had a snack just before”), slowness in eating (SE, example item 1: “My child takes more than 30 min to finish a meal”, example item 2: “My child eats more and more slowly during the course of a meal), food fussiness (FUS, example item 1: “My child is difficult to please with meals”, example item 2: “My child decides that s/he doesn’t like a food, even without tasking it”), and emotional undereating (EUE, example item 1: “My child eats less when angry”, example item 2: “My child eats less when s/he is tired”). Scores were averaged for each subscale, and mean subscale scores were used in the analyses.

#### 2.2.2. Sample Characteristics

Mothers reported demographic characteristics for themselves and their children, including maternal age, education (grouped as (partial) high school, partial college or specialized training, college graduate, or graduate training (masters, PhD)), pre-pregnancy BMI, and subjective social status (MacArthur Scale of Subjective Social Status, range from 1 to 10, with higher scores indicating higher subjective social status) [17], as well as child sex, race, and ethnicity. Children’s weights and heights were measured during the lab visits. Child BMI z-scores were calculated using the WHO Anthro version 3.2.2. [18], WHO Anthro Plus, and macros [19].

### 2.3. Data Analysis

Analyses were conducted using cross-sectional as well as longitudinal data. For the cross-sectional analysis, all mothers who had at least one observation on the Child Eating Behavior Questionnaire were included, using the first observation available. Cross-sectional Pearson correlations between appetitive trait scores and child age were examined first. For those Child Eating Behavior Questionnaire subscales where no significant Pearson (linear) correlation was seen with age, in a second step models were fit that tested for quadratic relationships. Given the wide age range in the current sample, non-linear relationships between age and child development might have been expected. For the longitudinal analysis, participants’ first and second observations on the Child Eating Behavior Questionnaire were selected. First, paired *t*-tests were run to determine age-related differences within individuals. Cohen’s d was used to determine effect sizes [20]. Next, Pearson correlation coefficients were used to examine tracking within individuals, i.e., to test whether children kept their relative rankings amongst the group from first to second observations (i.e., rank-order stability across assessments). To adjust for the child’s age at the first observation and also for the time lag between observations 1 and 2, two additional partial Pearson correlations were conducted: one adjusting for child age, the other adjusting for child age and time lag. Analyses were conducted using SPSS 27 (IBM Corp., Armonk, NY, USA).

## 3. Results

In total, 335 parents completed the Child Eating Behavior Questionnaire at least once while their children were between the ages of 2.0 and 15.2 years. Of these, 127 participants provided two observations, with an average time lag between measurements of 13.29 ± 3.80 months. Sample characteristics of the 335 participants are shown in Table 1.

### 3.1. Cross-Sectional Results

Cross-sectional Pearson correlations between appetitive trait scores and child ages are presented in Table 2. Correlations for Child Eating Behavior Questionnaire subscales indicated decreases in satiety responsiveness (r = −0.160, *p* = 0.004), slowness in eating (r = −0.266, *p* < 0.001), and desire to drink (r = −0.227, *p* < 0.001) with age and increases in emotional overeating (r = 0.203, *p* < 0.001) with age. Quadratic relationships were examined for food responsiveness, enjoyment of food, and food fussiness, since these showed non-significant linear relationships with child age. The former two were not significant (*p* = 0.213 and *p* = 0.660), while food fussiness showed a quadratic relationship (upside-down U-shape) with age, with a peak of fussiness around the age of 6 years (beta = −0.568, *p* = 0.017).

### 3.2. Longitudinal Results

Table 3 shows the mean scores of the Child Eating Behavior Questionnaire subscales at observation 1 and observation 2, as well as the results of the paired *t*-tests for age-related changes within individuals. Paired *t*-tests for Child Eating Behavior Questionnaire subscales indicated an increase in emotional overeating with age from the first to the second observation (M_1st_ = 1.55 vs. M_2nd_ = 1.69, *p* = 0.005). No significant mean change in appetitive traits between the first and second observations was seen for food responsiveness, enjoyment of food, desire to drink, satiety responsiveness, slowness in eating, food fussiness, or emotional undereating.

Table 4 outlines the results of paired correlations for tracking within individuals. All eight Child Eating Behavior Questionnaire subscales showed significant positive correlations across observations: food responsiveness (r = 0.644, *p* < 0.001), enjoyment of food (r = 0.708, *p* < 0.001), emotional overeating (r = 0.562, *p* < 0.001), desire to drink (r = 0.533, *p* < 0.001), satiety responsiveness (r = 0.726, *p* < 0.001), slowness in eating (r = 0.685, *p* < 0.001), food fussiness (r = 0.760, *p* < 0.001), and emotional undereating (r = 0.576, *p* < 0.001). The same pattern of positive correlations was seen when adjusting for child age at the first Child Eating Behavior Questionnaire observation or adjusting for child age at the first Child Eating Behavior Questionnaire observation and the time lag between the first and second Child Eating Behavior Questionnaire observations.

## 4. Discussion

The aim of this study was to investigate tracking and age-related differences in appetitive traits as assessed more narrowly in early to middle childhood, extending some of the previous studies conducted in Europe to children in the US. Cross-sectionally, we found that six out of the eight subscales assessed during early to middle childhood, were correlated with child age (i.e., emotional overeating, emotional undereating, desire to drink, satiety responsiveness, and slowness in eating), including one (food fussiness) showing a quadratic relationship and highlighting the importance of examining non-linear relationships. Longitudinally, we saw little evidence for age-related change within individuals, but we found that appetitive traits tracked within individuals. On the whole, our results demonstrated a decrease in food avoidant behaviors with age and an increase in emotional overeating with age, which is consistent with population survey and cohort data suggesting increasing likelihood of obesity development as children grow older [21].

Our cross-sectional observation that food avoidant traits were negatively associated with age was dependent on the appetitive dimension. The strongest age difference was for slowness in eating, which showed a negative correlation with age. However, significant negative correlations were also seen for satiety responsiveness and emotional undereating. A potential parsimonious explanation for these combined findings is that, while very young children are able to regulate their energy intake [22,23], this ability erodes over time due to environmental influences, including controlling parent feeding practices (such as parents’ restriction of children’s food intake) [24]. However, it is also possible, particularly within the infancy period, that eating speed increases along with motor dexterity and that as children get older they are increasingly involved in structured activities, such as childcare and school, that constrain schedules and effectively dictate how much time they spend eating, while exposure to social influences may lessen food avoidant behaviors. The decrease in emotional undereating could potentially have an additional set of determinants. For example, as children age, their emotion regulation may improve [25], making them less vulnerable to acute stress from extreme negative emotions, which may be more likely to decrease appetite than less severe emotions [26]. For food fussiness, a quadratic relationship with age was found, such that levels of food fussiness increased to about age six years before they started to decline. This finding is in line with those previously reported in a life-course analysis of pooled data from Ireland (N = 3246), where food neophobia (i.e., reluctance to eat novel foods) increased with age from 1 to 6 years before decreasing until early adulthood [27].

Our observation that emotional overeating increased with age was apparent in both our cross-sectional correlations with age and our within-subject paired *t*-tests using longitudinal data. While many appetitive characteristics have previously demonstrated strong genetic influence, emotional overeating has been found to show relatively high levels of environmental influence [28,29]. It is therefore possible that emotional overeating increases along with increases in potential environmental triggers. For example, as they progress through development, children are increasingly exposed to diverse palatable energy-dense foods as well as social situations that could promote emotional overeating. Although Farrow et al. [9] did not find a significant difference in emotional overeating between the ages of 2 and 5 years, our results are in line with several other previous studies that found that emotional overeating increases with age [8,30,31,32].

The continuity in appetitive characteristics that we observed in the majority of our within-subject tests (i.e., no significant mean differences in seven of the eight Child Eating Behavior subscales from the first to the second observation) was consistent with the results of Farrow and colleagues [9]. While Ashcroft et al. [8] and da Costa et al. [11] found discontinuity in all subscales they assessed, both our group’s and Farrow et al.’s results were consistent with continuity across observations (with one exception each: desire to drink (Farrow) and emotional overeating (present study)). Our study extends the latter’s findings by observing continuity for appetitive traits through to middle childhood. The different results between studies are likely due to there being less developmental change occurring from 2 to 5 years [9] or across 13 months (present study) than across a 7-year timespan from early childhood (age 4) to middle childhood (age 11) [8].

All eight appetitive characteristics showed tracking through childhood. That is, children who scored relatively high for these traits at their first observation in childhood (mean age: 6 years) maintained high scores relative to their peers at their second observation (approximately 13 months later). The Child Eating Behavior Questionnaire findings are largely in line with previous studies indicating strong positive associations across observations, even with varying time lags [8,9,33,34,35,36,37]. The stronger correlations observed in our study compared with the study by Ashcroft et al. [8] are likely due to the smaller time lag between our observations. Together with previous findings, our current results suggest that, like temperament [38], appetitive characteristics track through childhood.

### Strengths and Limitations

The findings of our study need to be considered in the light of its strengths and weaknesses. A feature of our study was that ages and time lags between observations varied across participants. While this variability could be viewed as a disadvantage, we note that our results hold even when controlling for this variation, promoting the generalizability of our results across ages and time lags. As shown in Table 1, more than half of the children had their first Child Eating Behavior Questionnaire observation before the age of 6 years. Another potential limitation was that children’s appetitive traits were reported by mothers using questionnaires, potentially leading to shared observer and shared method bias. However, any bias due to maternal report was likely consistent across both observations, and the Child Eating Behavior Questionnaire has been validated against behavioral tests [39]. Nevertheless, replication using repeated behavioral measures of appetitive characteristics obtained across a variety of settings would increase confidence in our results. It should also be noted that generalizability to other US families might be limited by the homogenous characteristics of the study sample, since the majority of the participants in our study (nearly 75%) identified as White. Finally, correlation coefficients between appetitive traits and age were small in this study, explaining only a limited amount of variance. Additionally, the Cohen’s d value for the mean change in emotional overeating was < 0.3, and the clinical significance of these results is unclear.

## 5. Conclusions

To conclude, we found in the current study that appetitive characteristics measured in both early and middle childhood are largely stable and continuous. Future research examining the stability and continuity of appetitive characteristics across different developmental stages, starting in infancy and incorporating several assessment timepoints, will be essential to more fully understand the development of appetite and how it is temporarily or more chronically impacted by environmental (e.g., parent feeding) or other influences (for a relevant conceptual model, see [38]). For example, individual differences in child characteristics at any one observation might be the result of individual variation in the rate of brain maturation or in the timing of genetic influences. Since personality traits are more stable in older adults than in children or young adults [38], it would also be of interest to examine development across the whole life course. Nonetheless, our existing results have implications. In particular, our robust finding of increased emotional overeating with age may suggest a target behavior that could be addressed in early life to limit obesity risk. For example, a recent study demonstrated that a responsive parenting intervention in early life decreased emotional overeating in children, and the effect of the intervention on emotional overeating was mediated by parents’ use of food to soothe the child, suggesting a potential parental behavior that could be targeted [40]. An increased understanding of appetite development could also provide foundations for further investigation of biological contributions to appetite development and inform the development of interventions for parents that aim to promote parent–child food-related interactions that represent the best “fit” between parental behaviors and the child’s unfolding appetitive tendencies.

## Figures and Tables

**Table 1:** Sample characteristics of participants completing the Child Eating Behavior Questionnaire.

		Child Eating Behavior Questionnaire (N = 335)		
	n	Mean (or N)	SD (or %)	Range
Mothers				
Maternal age	330	36.64	6.43	21.08 to 57.42
Maternal education	312			
(Partial) high school		32	10.3	
Partial college or specialized training		75	24.0	
College graduate		86	27.6	
Graduate training (master’s, PhD)		119	38.1	
MacArthur Scale of Subjective Social Status [17]	313	5.51	1.59	2 to 10
Maternal pre-pregnancy BMI	309	26.6	6.88	16.6 to 53.0
Children				
BMI z-score (WHO reference data)	275	0.40	1.32	−4.71 to 5.14
Sex (female)	335	143	42.6	
Race	323			
Asian		4	1.2	
Black or African American		18	5.6	
More than 1 race		59	18.3	
White		242	74.9	
Ethnicity	328			
Hispanic/Latino		54	16.5	
Non-Hispanic/Latino		274	83.5	
Age in years at 1st observation (all participants)	335	6.04	2.98	2.00 to 15.24
2–3 years		105	31.3	
4–5 years		79	23.6	
6–7 years		66	19.7	
8–9 years		41	12.2	
10+ years		44	13.1	
Age in years at 1st observation (participants with 2 observations)	127	5.86	2.68	2.01 to 12.45
Age in years at 2nd observation	127	6.97	2.69	2.53 to 13.46
Time lag between 1st and 2nd observations (in months)	127	13.29	3.80	4.87 to 32.74

**Table 2:** Pearson correlations between child appetitive traits assessed with the Child Eating Behavior Questionnaire and child age.

Child Eating Behavior Questionnaire	Child Age	
	r	*p*
FR	−0.055	0.319
EF	−0.073	0.187
EOE	0.203	<0.001
DD	−0.227	<0.001
SR	−0.160	0.004
SE	−0.266	<0.001
FUS	−0.014	0.797
EUE	−0.119	0.031

Abbreviations: FR = food responsiveness, EF = enjoyment of food, EOE = emotional overeating, DD = desire to drink, SR = satiety responsiveness, SE = slowness in eating, FUS = food fussiness, EUE = emotional undereating.

**Table 3:** Paired *t*-tests to determine age-related changes within individuals for Child Eating Behavior Questionnaire subscales.

	All Participants (1st Observation)			Observation 1		Observation 2		Paired *t*-Test			
	Cronbach’s Alpha	Mean	SD	Mean	SD	Mean	SD	Mean Change (SD) Ob2–Ob1	*t*-Score	*p*-Value	Cohen’s d
Child Eating Behavior Questionnaire	N = 337			n = 127							
FR	0.762	2.34	0.76	2.30	0.73	2.26	0.72	−0.04 (0.61)	−0.698	0.486	−0.062
EF	0.862	3.74	0.73	3.74	0.74	3.70	0.73	−0.04 (0.56)	−0.799	0.426	−0.072
EOE	0.744	1.59	0.57	1.55	0.54	1.69	0.63	0.14 (0.55)	2.829	0.005	0.251
DD	0.841	2.71	1.02	2.57	1.01	2.54	1.00	−0.03 (0.97)	−0.338	0.736	−0.030
SR	0.742	2.87	0.64	2.94	0.61	2.97	0.61	0.03 (0.45)	0.791	0.431	0.071
SE	0.770	2.84	0.79	2.93	0.80	2.96	0.81	0.04 (0.64)	0.626	0.532	0.056
FUS	0.915	2.90	0.91	2.89	0.84	2.89	0.90	−0.01 (0.61)	−0.153	0.879	−0.014
EUE	0.809	2.51	0.87	2.56	0.81	2.57	0.88	0.01 (0.78)	0.172	0.864	0.015

Abbreviations: FR = food responsiveness, EF = enjoyment of food, EOE = emotional overeating, DD = desire to drink, SR = satiety responsiveness, SE = slowness in eating, FUS = food fussiness, EUE = emotional undereating.

**Table 4:** Paired correlations between observations 1 and 2 to determine tracking within individuals for Child Eating Behavior Questionnaire subscales.

	Child Eating Behavior Questionnaire (n = 127)					
	Pearson Correlation		Partial Correlation with Age at 1st Observation		Partial Correlation with Age at 1st Observation and Time Lag	
	r	*p*-Value	r	*p*-Value	r	*p*-Value
FR	0.644	<0.001	0.644	<0.001	0.648	<0.001
EF	0.708	<0.001	0.708	<0.001	0.702	<0.001
EOE	0.562	<0.001	0.550	<0.001	0.555	<0.001
DD	0.533	<0.001	0.524	<0.001	0.518	<0.001
SR	0.726	<0.001	0.724	<0.001	0.722	<0.001
SE	0.685	<0.001	0.679	<0.001	0.689	<0.001
FUS	0.760	<0.001	0.763	<0.001	0.767	<0.001
EUE	0.576	<0.001	0.574	<0.001	0.576	<0.001

Abbreviations: FR = food responsiveness, EF = enjoyment of food, EOE = emotional overeating, DD = desire to drink, SR = satiety responsiveness, SE = slowness in eating, FUS = food fussiness, EUE = emotional undereating.

## Data Availability

Data will be made available by the corresponding author upon reasonable request.
